# Radiation makes cells select the form of death dependent on external or internal exposure: apoptosis or pyroptosis

**DOI:** 10.1038/s41598-023-38789-0

**Published:** 2023-07-25

**Authors:** Kazuko Shichijo, Toshihiro Takatsuji, Darkhan Uzbekov, Nailya Chaizhunusova, Dariya Shabdarbaeva, Minako Kurisu, Yoshio Takahashi, Valeriy Stepanenko, Almas Azhimkhanov, Masaharu Hoshi

**Affiliations:** 1 Department of Tumor and Diagnostic Pathology, Atomic Bomb Disease Institute, Nagasaki University, 1-12-4 Sakamoto, Nagasaki, 852-8523 Japan; 2 Nagasaki University, 1-14 Bunkyo, Nagasaki, 852-8521 Japan; 3 School of Medicine, Dokkyo Medical University, 880 Kitakobayashi, Mibu, Shimotsugagun, Tochigi 321-0293 Japan; 4 Department of Pathological Anatomy and Forensic Medicine, Semey State Medical University, Abay Str., 103, Semey, 071400 Kazakhstan; 5 Department of Earth and Planetary Science, Graduate School of Science, The University of Tokyo, 7-3-1 Hongo, Bunkyo-ku, Tokyo, 113-0033 Japan; 6 Research Institute for Marine Resources Utilization, Japan Agency for Marine-Earth Science and Technology, 2-15 Natsusima-cho, Yokosuka-shi, Kanagawa 237-0061 Japan; 7 A.Tsyb Medical Radiological Research Center—National Medical Research Center of Radiology, Ministry of Health of Russian Federation, 249036 Obninsk, Russia; 8 National Nuclear Center of the Republic of Kazakhstan, Beibyt atom st., 2B, Kurchatov, 071100 Kazakhstan; 9 The Center for Peace, Hiroshima University, Higashi-senda-machi, Naka-ku, Hiroshima, 730-0053 Japan

**Keywords:** Disease model, Cell biology, Pathogenesis

## Abstract

Internal radiation exposure from neutron-induced radioisotopes environmentally activated following atomic bombing or nuclear accidents should be considered for a complete picture of pathologic effects on survivors. Acute and localized high dose radiation exposure from hot particles taken into the body must induce cell death and severe damage to tissues, whether they are proliferating or not. However, very little the cellular and molecular mechanisms underlying this internal radiation pathology has been investigated. Male Wistar rats were internally exposed to ^56^MnO_2_ powder by inhalation. Small intestine samples were investigated by histological staining at acute phase (6 h, 3 days and 14 days) and late phase (2, 6 and 8 months) after the exposure. Histological location and chemical properties of the hot particles embedded in small intestinal tissues were analyzed by synchrotron radiation—X-ray fluorescence—X-ray absorption near-edge structure (SR–XRF–XANES). Hot particles located in the intestinal cavity were identified as accumulations of Mn and iron. Pathological changes showed evidence of crypt shortening, massive cell death at the position of stem cell zone, including apoptosis and pyroptosis from 6 h through 8 months in the internal exposed rats.

## Introduction

Diarrhea caused by radiation is generally referred to as a high radiation dose effect on the gastrointestinal (GI) tract when the external exposure is 10 Gy or more^1–3^, which is a radiation lethal dose that is far exceeds the dose which will kill 50% of the exposed population within 60 days (LD50/60, about 3 Gy) without treatment such as bone marrow transplantation etc., the patient would be dead anyway^4^.

On the other hand, there is a group of Hibakusha not in the city but moved to these cities soon after the detonations (called A-bomb indirect exposed survivor), externally exposed radiation dose was considered to be nearly 0, and have lived with acute radiation syndromes like diarrhea. The incidence rate of diarrhea has been reported to be 50%^5^. If high-dose radiation injury occurred in the small intestine, which is the second most radiosensitive organ after bone marrow cells, it could be attributed to localized high-dose radiation exposure around ingested radioactive materials.

We have already demonstrated internal exposure experiments and reported that internal exposure to rat lungs samples causes local ultra-high doses of radiation, resulting in severe damage not seen with external exposure^6^.

In the present study, we investigated whether internal exposure of rat small intestines samples can cause severe small intestinal damage as associated with local ultra-high doses. Moreover, we present X-ray spectroscopic imaging data of an intestinal tissue sample taken in the area of maximal concentration of Mn. The method employed here was synchrotron radiation X-ray fluorescence mapping using X-ray microbeam (SR-XRF) with identification of Mn chemical species at the point of interest by X-ray absorption near-edge structure (XANES) spectroscopy. The results showed that ^56^MnO_2_ particles were embedded and located in the small intestine cavity, and injured tissue with DNA double-strand brake (DSB) at the stem cell position until 8 months.

Radiation originated from radioactive materials in soil, dust and others may give a significant effect of radiation on the atomic bomb survivors of Hiroshima and Nagasaki, as well as those affected by nuclear power plant accidents, for example the Fukushima nuclear accident, and other nuclear disasters that scattered radioactive particles called “hot particles”^7,8^, in addition to the initial radiation directly received from the bombs or other sources. This may be more important for the risk assessment to the people in the future.

^56^Mn is known to be one of the dominant radioisotopes produced in soil by neutrons from the bomb (DS86 Vol. 1^9^). Due to its short physical half-life of 2.58 h, ^56^Mn emits radiation during substantially only the first few hours after inhalation. We investigated the biologic effects of internal radiation exposure after inhalation by ^56^MnO_2_ powder on rats’ lung, compared with group exposed to γ-ray externally^6,10–13^. Prolonged, and severe acute and late radiation injury effects were observed in the lungs of the ^56^MnO_2_ internal exposure group, while the external γ-ray whole-body exposure group showed mild effects^6^, led us to a need to determine initial effects of the early exposure event including target cell lineage.

The intestinal tract is one of the most important organs causing severe radiation injury. Small intestine is one of the organs most susceptible to radiation injury. Radiation is the cause of radiation-induced diarrhea and radiation sickness, and intestinal death occurs in humans exposed to high-dose (> 10 Gy) whole body radiation exposure^1^. Interestingly, in our series of internal radiation exposure experiments, the tissue average accumulated absorbed dose of small intestine was the second highest after the large intestine among the 12 organs; liver, heart, kidney, tongue, lungs, esophagus, stomach, small intestine, large intestine, trachea, eyes, skin, we examined^14–16^. These suggest that peoples who inhale or ingest dust containing radioactive particles may be similarly exposed to high radiation doses in digestive system.

**Figure 1:**
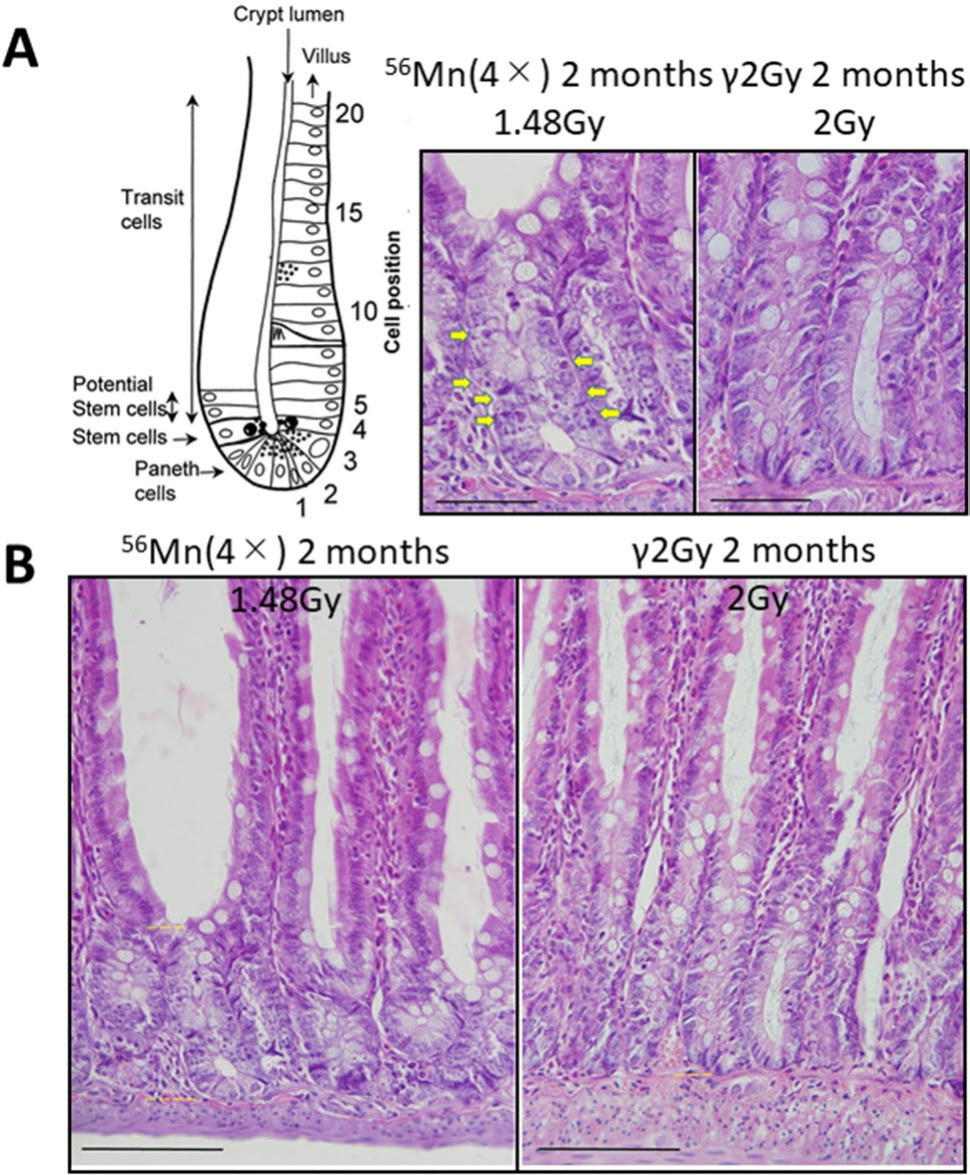
Apoptosis or pyroptosis in rat small intestine after internal exposure is occurring extremely different crypt cell position (**A**). Crypt shortening occurred in internal exposure (**B**). These most likely correlate to the continued cell damage observed even beyond 2 months. Arrows indicate apoptotic or pyroptotic cells. (**A**) Cell position of small intestine (Potten^17^). Crypt damage 2 months after 4× ^56^Mn internal exposure and after γ2Gy external exposure. (**B**) Crypt shortening 2 months after 4× ^56^Mn internal exposure and after γ2Gy external exposure. H&E stain. ×100. Scale bar: 50 µm (**A**) ×40. Scale bar: 100 µm (**B**).

Here we present early stage effects at 6 h and 3, 14 days after exposure of 3 activation levels (1×, 2×, 4×, see “Materials and methods”) of ^56^MnO_2_ powder and late stage effects at 2, 6 and 8 months after high internal exposure to the small intestine with anatomically known stem cell location and epithelial turnover rate of 3 days^17^ (Fig. 1). Understanding possible potential late effects of early stage internal β-ray exposure with ^56^Mn, which has a short physical half-life, after a successive epithelial cell turnover, leads to a more complete picture of the pathological effects of internal radiation in the small intestine.

## Results

### Early event damage to small intestinal tissue for internal exposure is uncharacteristically rapid and severe at the stem cell position

The average radiation doses of each organ received in ^56^Mn(4×) and ^56^Mn(2×) groups were substantially almost same or lower than those received in ^56^Mn(1×) group. The tissue average accumulated absorbed doses in the small intestine were 1.33 ± 0.17 Gy in ^56^Mn(1×), while 1.48 Gy ± 0.37 in ^56^Mn(4×) group and 0.58 Gy ± 0.15 in ^56^Mn(2×) group^6^ (Table 1, Supplementary Table S2) at the time the activity of ^56^Mn (half-life 2.58 h) were almost totally attenuated (3 days, 14 days, 2 months, 6 months, 8 months). The doses of Hour 6 are 80% of the doses.

**Table 1:** Tissue accumulated absorbed doses^6^ and ratio of crypt vs. villous length per crypts (Individual data are listed in Supplementary Table S2) of the small intestine in rats exposed to ^56^Mn and γ2Gy.

Groups	Tissue absorbed doses^6^	Ratio of crypt vs. villous length				
	Gy	8 months				
^56^Mn(1×)	1.33 ± 0.17	0.388(101) ± 0.006				
Mn-stable	0 ± 0	0.381(99) ± 0.005				
γ2Gy	2 ± 0	0.348(90) ± 0.005^b^				
Control	0 ± 0	0.381(99) ± 0.002				

Groups	Gy	6 h	3 days	14 days	2 months	6 months
^56^Mn(4 ×)	1.48 ± 0.37	0.340 (88) ± 0.017^a,e^	0.275 (71) ± 0.007^b,d^	0.303 (78) ± 0.010^b^	0.280 (72) ± 0.008^b,d^	0.384 (99) ± 0.006
^56^Mn(2×)	0.58 ± 0.14	0.403 (104) ± 0.019	0.280 (72) ± 0.009^b,d^	0.303 (78) ± 0.008^b,c^	0.306 (79) ± 0.012^a,c^	0.380 (98) ± 0.012
γ2Gy	2 ± 0	0.386 (100) ± 0.015	0.413 (107) ± 0.008	0.342 (89) ± 0.011^a^	0.364 (94) ± 0.006	0.362 (94) ± 0.008^a^
Control	0 ± 0	0.386 (100) ± 0.016	0.374 (97) ± 0.005	0.386 (100) ± 0.006	0.386 (100) ± 0.009	0.388 (100) ± 0.009

The doses of ^56^Mn are for 3 days, 14 days, 2 months, 6 months, 8 months. The doses of 6 hours are 80% of the doses.

Mean ± SE, (%) compared with 0 h, ^a^p < 0.05, ^b^p < 0.01, vs Control. ^c+^p < 0.05, ^d++^p < 0.01 vs. γ2Gy. ^e^p < 0.01, vs ^56^Mn(2×). N = 3–4.

**Figure 2:**
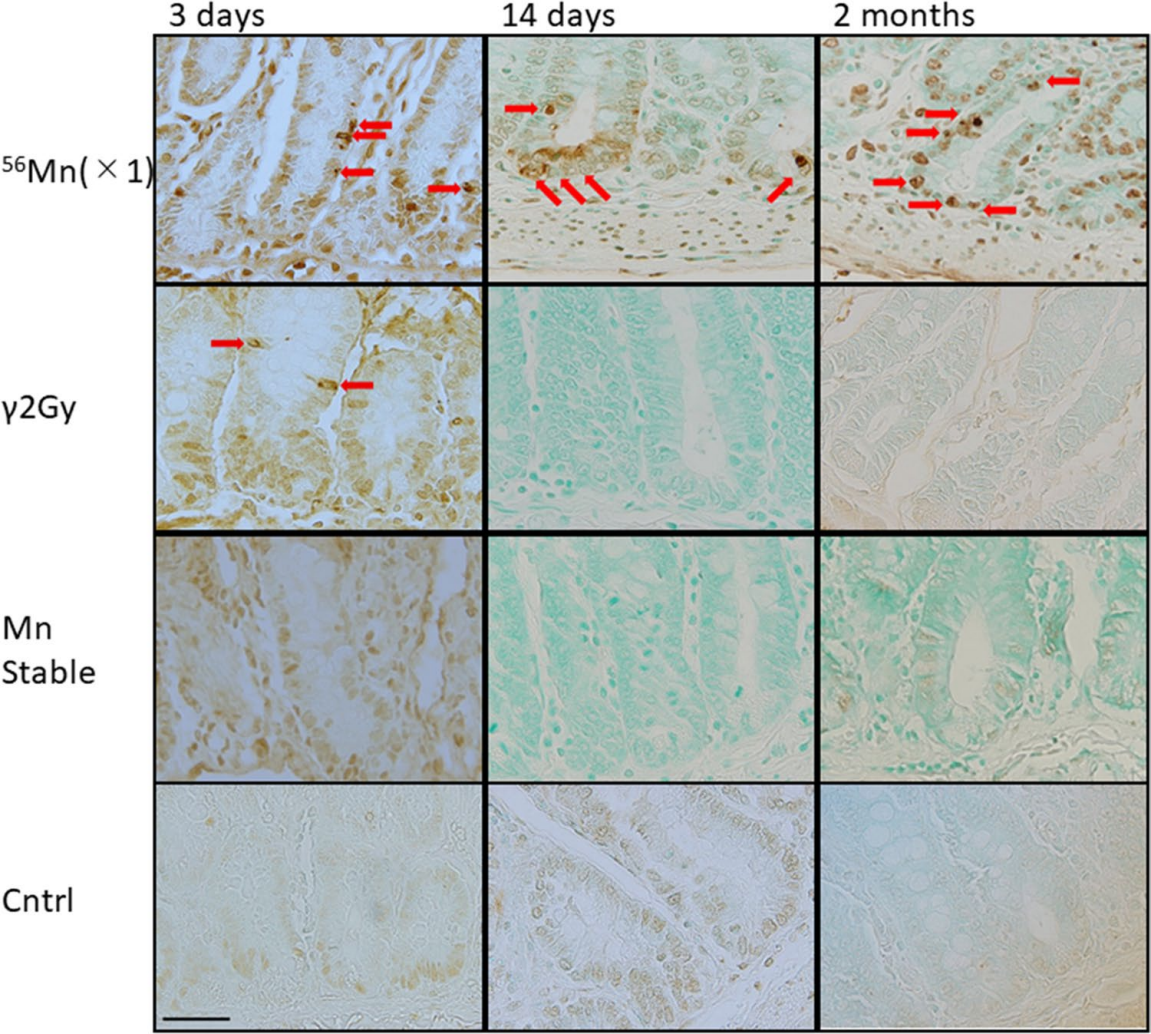
Apoptosis by TUNEL method. Apoptosis was scattered in the small intestine of rats at the position of transit cell 3 days after 1× ^56^Mn internal exposure. Apoptosis was observed in the small intestine of rats mainly at the position of stem cell 14 days after ^56^Mn(1×) internal exposure. Apoptosis observed in small intestine of rats at the position of transit cell and stem cell 2 months after 1× ^56^Mn internal exposure. No change was observed in the Mn stable group and the control group except that the expression of apoptosis was slightly increased at the position of transit cell in the γ2Gy external exposure group on the 3 days after exposure. Arrows indicate TUNEL positive cells. ×100. Scale bar: 50 µm.

After ^56^Mn(1×) internal exposure, apoptosis stained by TUNEL method was observed scattering in the small intestine of rats at the position of transit cell at 3 days and mainly at the position of stem cell at 14 days, no change was observed in the Mn stable group, the unexposed control group and the γ2Gy externally exposed group except that the expression of apoptosis was slightly increased at the position of transit cell in the externally exposed group on the 3rd day (Fig. 2).

**Figure 3:**
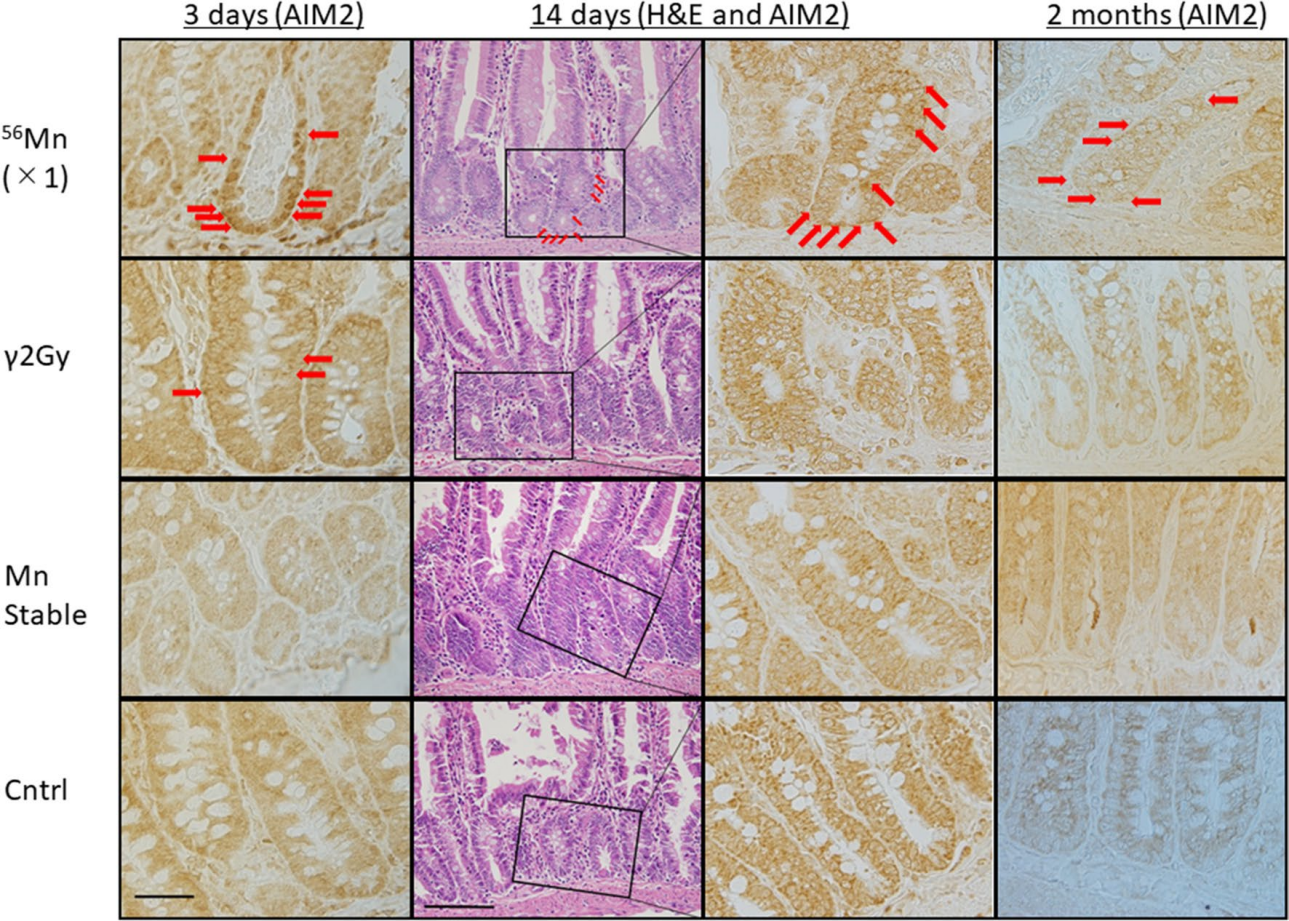
Pyroptosis by AIM2 immunohistochemistry. Pyroptosis was scattered in the small intestine of rats at the position of stem cell and transit cell 3 days after 1× ^56^Mn internal exposure. Pyroptosis observed in small intestine of rats in the position of stem cell and transit cell 14 days after 1× ^56^Mn internal exposure. At 14 days, a “non-straight and distorted crypts” structure was formed and structural abnormality of crypt was observed (×40, H&E, Scale bar: 100 µm). Pyroptosis observed in small intestine of rats in the position of stem cell and transit cell 2 months after 1× ^56^Mn internal exposure. No change was observed in the Mn stable group and the control group except that the expression of AIM2 was slightly increased in the γ2Gy external exposure group on the 3 days after exposure. Arrows indicate AIM2 positive cells. ×100, Scale bar: 50 µm.

**Figure 4:**
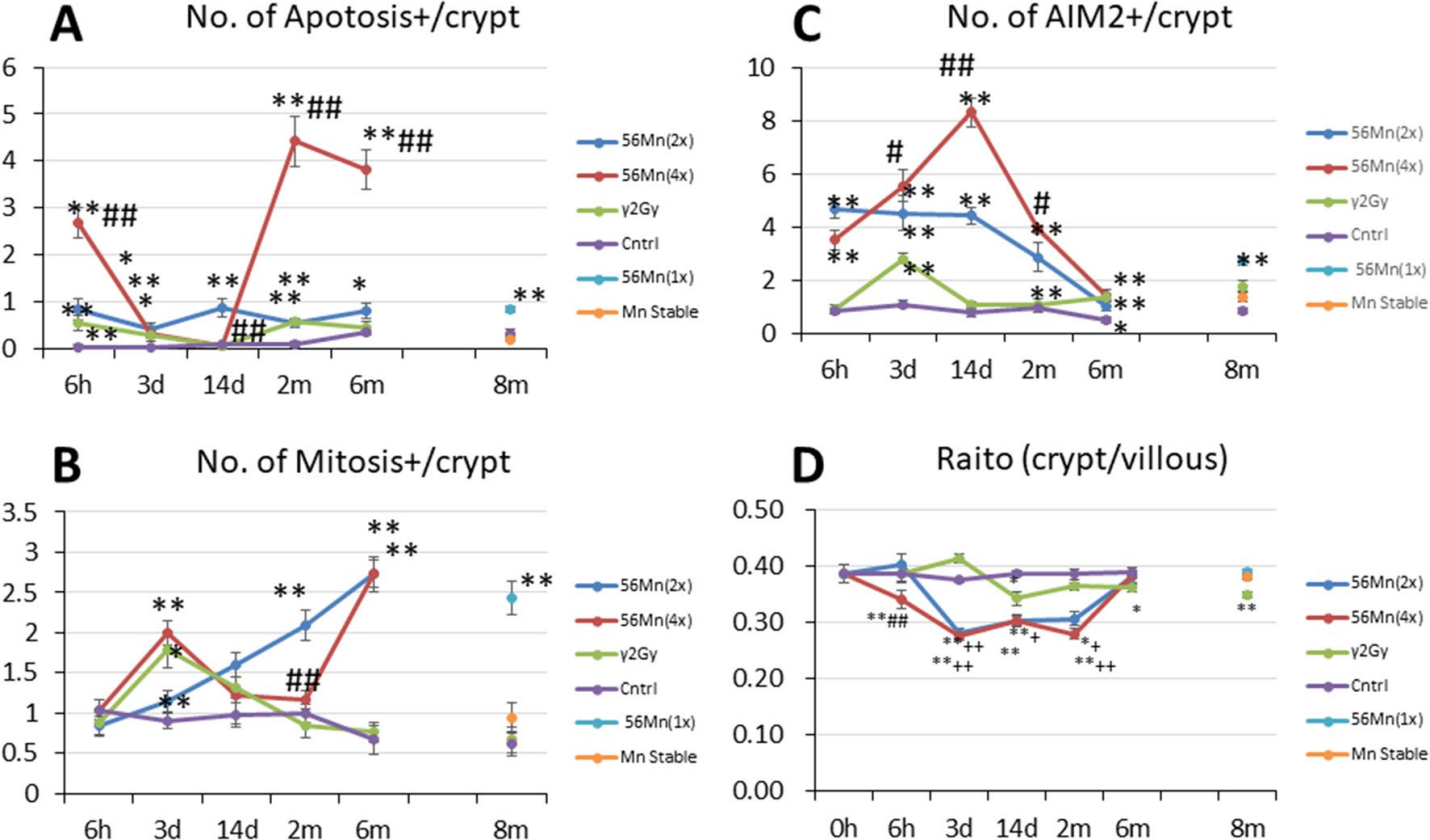
Histological scored findings in the small intestine in rats of internally exposed to Mn stable and ^56^Mn(1×, 2×, 4×), of externally exposed to 2.0 Gy γ (γ2Gy) and of control groups. (**A**) shows apoptosis findings of ^56^Mn(2×), ^56^Mn(4×), γ2Gy, control, ^56^Mn(1×) and Mn stable, and (**B**) shows findings for mitosis for the same experiments. (**C**) shows the AIM2 findings for the same experiments, and (D) shows the Raito of crypt/villous findings for the same experiments. Bars, mean ± SE (*n* = 3–6). *p < 0.05, **p < 0.01 vs. Control. ^+^p < 0.05, ^+ +^ p < 0.01 vs. γ2Gy. ^#^p < 0.05, ## p < 0.01 vs. ^56^Mn(2×). The experimental data of this study is shown in Supplementary Table S1.

Pyroptosis observed after ^56^Mn(1×) internal exposure detected by absent in melanoma 2 (AIM2) staining prominently scattered at the positions of stem and transit cell in the small intestine, from top to bottom of the crypt, AIM2-positive cells were stacked together to form a mass at 3 days and in the same positions at 14 days. Aberrant crypt was formed and structural abnormality of crypt was observed at 14 days by H&E stain. AIM2-positive abnormal crypts (non-straight and distorted crypts) were formed as structural abnormalities. AIM2-positive abnormal crypts are not seen in the γ2Gy external exposure whereas the dose is about the same of the ^56^Mn internal exposures on average (Fig. 3). The internally exposed groups had consistently higher levels of AIM2 expression compared to the externally exposed groups and unexposed control. AIM2 expression in ^56^Mn(2×) exposed and ^56^Mn(4×) exposed groups peaked at 6 h and 14 days post-exposure, respectively, whereas it increased slightly on 3 days and returned on 14 days in the externally exposed group. In the internally exposed group, AIM2 expression was significantly higher in the ^56^Mn(4×) group than in the ^56^Mn(2×) group at stem cell-rich-locations from 3 days through 2 months after exposure. No change was observed in the Mn stable group and unexposed control group (Fig. 4C, Supplementary Table S1).

The histological changes in the small intestine of the γ2Gy externally exposed group showed slight increase in apoptosis at 6 h, an increase in mitosis at 3 days, slight increase in pyroptosis at 3 days and slight decrease in crypt length at 14 days. In contrast, in the ^56^Mn(4×) internally exposed group, apoptosis increased prominently at 6 h and slightly increased at 3 days, increase of mitosis was similar to the γ2Gy externally exposed group at 3 days, pyroptosis increase from 6 h through 6 months in ^56^Mn(4×) group (Figs. 4, Supplementary Table S1), and crypt length (ratio of crypt/villous) severely decreased from 6 h through 2 months, which was less pronounced in ^56^Mn(2×) group (Table 1, Supplementary Table S2, Figs. 1B, 4D, Supplementary Table S1). At 14 days in ^56^Mn(1×) group, a morphologically abnormal crypts with intense pyroptosis were found, where was formed a “non-straight and distorted crypts” structure clearly observed by H&E stain. Slight hyperemia was also observed in the interstitium (Fig. 3).

To compare the effect of internal and external radiation on radiation-induced injury in the rat small intestine, ratio of crypt vs. villous length per crypts were measured in samples taken from 0 to 8 months after exposure (Table 1, Supplementary Table S2). The ratio in internally exposed rats showed prominently decreases at 6 h (no lower, 87% lower), 3 days (72%, 71% lower) and 14 days (78%, 78% lower) in ^56^Mn(2×) and ^56^Mn(4×) groups, respectively, while the ratio in externally exposed γ2Gy rats slightly decreased at 14 days (89% lower). For groups internally exposed with ^56^Mn, the crypts were significantly shorter (crypt shortening) than externally exposed or unexposed control groups after 6 h in ^56^Mn(4×), 14 days in ^56^Mn(2×) and ^56^Mn(4×) (Figs. 1A, 4D, Supplementary Table S1). These results indicate that internal exposure causes significant morphological acuteradiation-induced injury to the small intestine of rats.

### Late histology with prominent apoptosis and pyroptosis in the small intestine at the wide range position including stem cell and transit cell for 1× 2× and 4× ^56^Mn exposures, with DNA damage response (Figs. 1, 2, 3, 4, 5, Supplementary Table S1).

**Figure 5:**
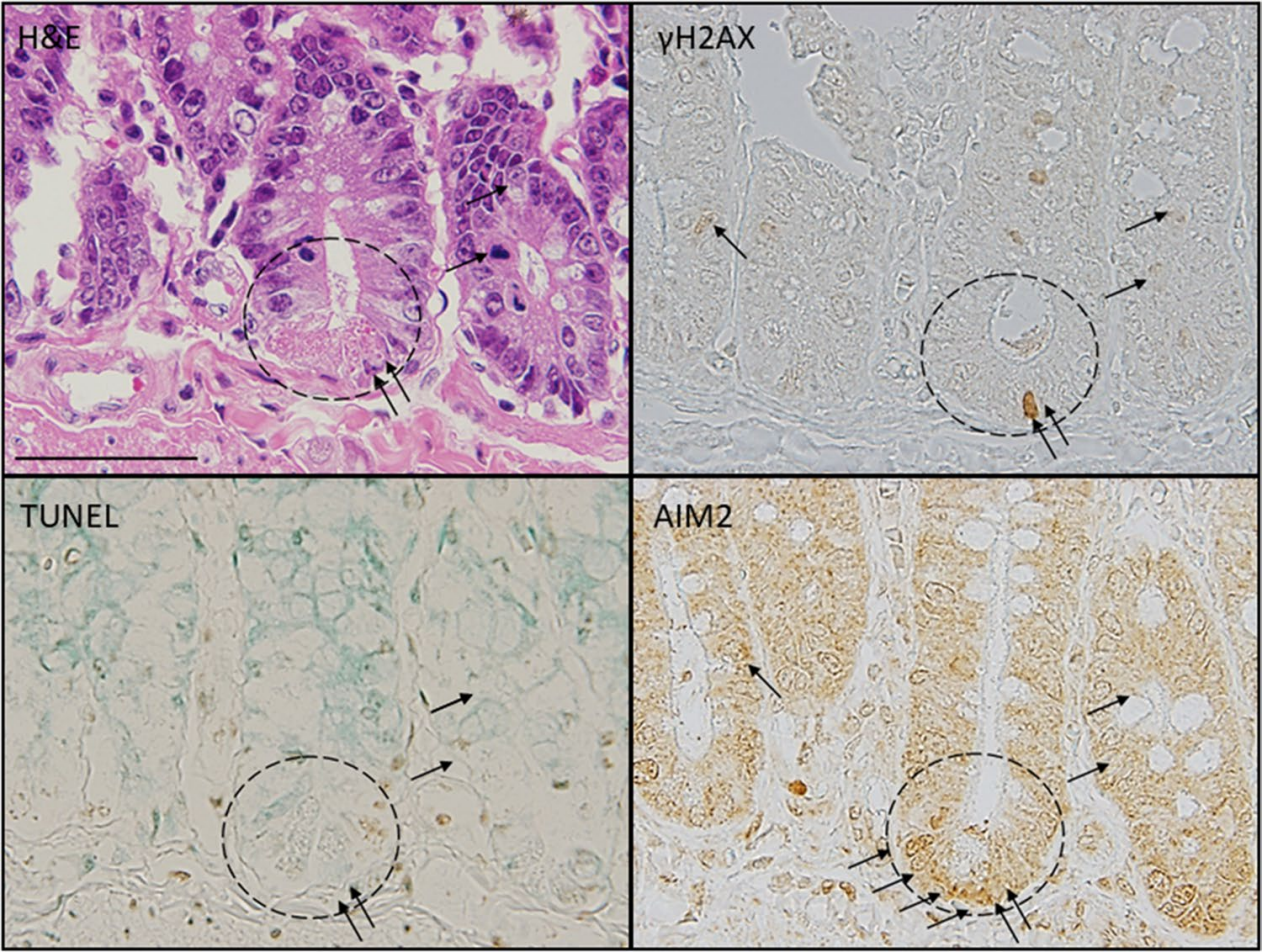
Apoptosis by TUNEL method, DNA DSB damage response by γ-H2AX and pyroptosis by AIM2 immunohistochemistry were observed in small intestine of rats in the position of stem cell 2 months after 4× ^56^Mn internal exposure. Pyroptosis, DNA-induced clustering of AIM2 protein caused pyroptotic cell death with characteristic plasma membrane swelling in small intestinal cells including the wide range of stem cell positions (H&E stain). Apoptosis was not so obviously observed in the position of stem cell (TUNEL). DNA DSB damage response by nuclear and cytoplasmic γ-H2AX expression clearly observed in the position of stem cell (γ-H2AX). Pyroptosis by AIM2 cytoplasmic expression was observed in the wide range of positions of stem cell (AIM2). No change was observed in the Mn stable group and the control group except that the expression of AIM2 was slightly increased in the γ2Gy external exposure group on the 3 days after exposure (Supplementary Fig. S1). Corresponding serial sections stained with H&E, TUNEL, γ-H2AX and AIM2. Arrows indicate positive cells. ×100. Scale bar: 50 µm.

**Figure 6:**
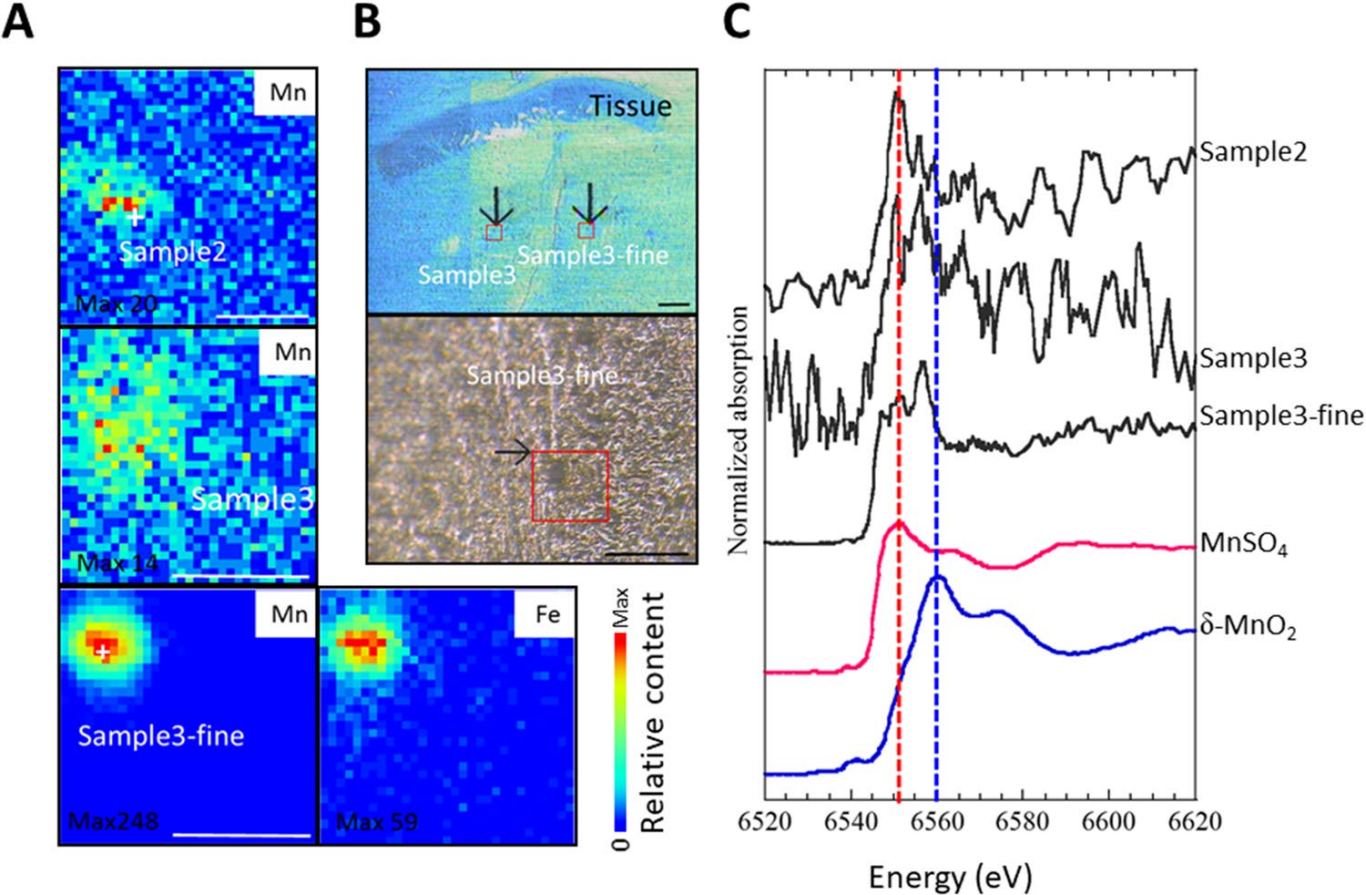
Early elementary profile, histopathologic image and Mn K-edge XANES spectra in small intestine tissue 6 h after 2× ^56^Mn internal exposure. (**A**) Elementary imaging (beam size: 1 µm × 1 µm) of Sample 2 (5 μm step, 140 × 140 μm), Sample 3 (5 μm step, 90 × 90 µm) and Sample 3-fine (5 μm step, 90 × 90 µm) of boxed areas marked in (**B**) are shown. (**C**) Mn K-edge XANES spectra, located in the small intestine cavity (Sample 2, Sample 3, Sample 3-fine). The Mn K-edge XANES spectra all of Sample 2, Sample 3 and Sample 3-fine were different from that of MnO_2_, but similar to that of MnSO_4_ solution. The red dotted lines indicate locations of peaks observed in MnSO_4_ solution. The special similarities indicate that Mn is not in the Mn^4+^ (Mn reagent, MnO_2_), but is Mn^2+^(MnSO_4_). Scale bar: 50 µm (**A**), 200 µm (**B** up), 100 µm (**B** low).

Apoptosis was observed in small intestine of rats at the position of transit cell and stem cell 2 (Fig. 2), 6 and 8 months after internal exposure. Mitosis increased in ^56^Mn(2×) at 2 months and 6 months, later in ^56^Mn(4×) at 6 months, and in ^56^Mn(1×) at 8 months (Fig. 4A, Supplementary Table S1). AIM2 expression was observed at the position of stem cell and transit cell 2 months after ^56^Mn(1×) internal exposure (Fig. 3), and higher level of pyroptosis, which persisted from 6 h onwards, was observed in activation level dependent manner for internally exposed groups of ^56^Mn(2×) and ^56^Mn(4×) (Fig. 4C, Supplementary Table S1), while no change was observed in the Mn stable group and the unexposed control group, and in the γ2Gy external exposure group, apoptosis and pyroptosis were slightly increased at 2 and 6 months, respectively (Fig. 4, Supplementary Table S1).

Severe crypt shortening, a morphological marker, was evident at 2 months both in groups of ^56^Mn(2×) (79% lower) and ^56^Mn(4×) (72% lower), while was slight at 6 and 8 months in the γ2Gy group (94%, 90% lower) (Table 1, Supplementary Table S2, Figs. 1B, 4, Supplementary Table S1).

From histological scored findings in Fig. 4, Supplementary Table S1, early effects of internal exposure foreshadowed the warning signs of late effects. High score levels for apoptosis, mitosis and pyroptosis, with prominent crypt shortening at 2× and 4× ^56^Mn exposure at 6 h continued to 8 months after irradiation. There were no pathological findings continued in the γ2Gy group, except for a small decrease in crypt shortening (94, 90%) after 6 and 8 months (Table 1, Supplementary Table S2, Fig. 4, Supplementary Table S1).

From the corresponding serial sections stained with H&E, phosphorylated histone H2AX (γ-H2AX) and AIM2 immunohistochemistry, and TUNEL method, pyroptosis was clearly detected by H&E stain and AIM2 protein expression in ^56^Mn(4×) group at 2 months. Apoptosis was not so obviously observed in the position of stem cell. DNA damage response detected by γ-H2AX expression clearly observed in the position of stem cell. Pyroptotic cell death was observed with DNA-induced clustering of AIM2 protein expression and with characteristic plasma membrane swelling in small intestinal cells including the wide range of transit and stem cell positions 2 months after 4× ^56^Mn internal exposure (Figs. 1A, 5).

### SR-XRF analysis revealed that masses of manganese and iron located in the small intestine cavity

Early elementary profile and histopathologic image were shown in small intestine tissue 6 h after 2× ^56^Mn internal exposure. Sample2, Sample3 and Sample3-fine were identified as condensed accumulations of manganese and iron (Fig. 6A,B). From the elemental distribution, we estimated the elementary profile for Sample2, Sample3 and Sample3-fine. The focus of Sample3-fine is a condensed accumulation of manganese and iron. It was located in the small intestine cavity. The somewhat anomalous presence, extra-tissue luminal side of these inhaled particles may have originated from a larger mass of manganese and iron in the process of digestive excretion with food or other matter. Fe elements were extremely abundant in sample3-fine in the small intestine, as in shot1 in the lungs, located in the bronchiole cavity^6^.

### XANES spectroscopy for the analysis of particles embedded in small intestine tissue samples (Sample2, Sample3 and Sample3-fine)

Particles embedded in small intestinal tissue samples (Sample2, Sample3 and Sample3-fine in Fig. 6A,B) were analyzed by XANES spectroscopy and found to be Mn; XANES spectroscopy shows the absorption energy position and intensity of Mn compounds, so these samples were metabolized Mn. Mn particles with a 4-valent chemical species as MnO_2_ changed to 2-valent when deposited in small intestinal tissue, as in lung tissue^6^ (Fig. 6C).

## Discussion

In small intestine of rat in ^56^Mn internal exposure groups (^56^Mn(1×), ^56^Mn(2×), ^56^Mn(4×)), crypt shortening until 2 months and pyroptosis until 8 months were observed more prominently than γ2Gy ^60^Co external exposure group. Tissue average absorbed dose of ^56^Mn(1×), ^56^Mn(2×) and ^56^Mn(4×) are 1.33 Gy, 0.58 Gy and 1.48 Gy respectively. (We will note as ^56^Mn(1×);1.33 Gy, ^56^Mn(2×); 0.58 Gy, ^56^Mn(4×); 1.48 Gy). The internal exposure, moreover, prevalently resulted in an increase of apoptosis, mitosis and genomic instability such as DNA DSB at the position prominently including stem cells at 2 months. Their stem cells may initially increase their proliferation by delayed arrest (stem cell exhaustion) and re-proliferation (cancer predisposition). Our results indicated an increasing possibility of digestive system failure and morbidity from the internal exposure.

At external doses of 12 Gy and above, the mortality rate of GI syndrome exceeds that of hematopoietic syndrome^1^. Radiation induces loss of intestinal crypts and disruption of the mucosal barrier. These changes cause abdominal pain, diarrhoea, nausea and vomiting and render the patient infectious. However, the cellular targets of GI syndrome and the mechanisms of radiation-induced cell death remain controversial.

We have already reported that (1) after 5 Gy, 50% lethal dose, whole body external radiation exposure, the mucosal length of the small intestine was 85% lower at 3 day but returned normal level at 7 day^18^ and that (2) after 8 Gy, 100% lethal dose, whole body external radiation exposure, the crypt length of the small intestine was 63% lower at 3 day but returned normal level at 5 day^19^. These results of high dose; 5 Gy and 8 Gy external exposure experiments were similar to the pathological findings at 6 h, 3 days and 14 days (early) and 2 months (late) after ^56^Mn internal exposure in our study, namely the crypt shortening (71% lower) at 3 day, but returned normal level at 6 months. Compared to 5 Gy external exposure, the ^56^Mn internal exposure of 2× and 4× in our study resulted in similar or more severe late pathological findings, crypt shortening by delayed arrest (stem cell exhaustion) and AIM2 expression of indicator of pyroptosis.

Although AIM2 is an innate immune sensor^20,21^, Hu B et al.^22^ found that AIM2-deficient mice were protected from lethality and intestinal damage caused by lethal doses of subtotal body irradiation (SBI). They showed that endogenous AIM2 formed nuclear punctures upon irradiation and considerable co-localization with γ-H2AX-positive foci was observed in the nucleus, suggesting that radiation can mobilize AIM2 to dsDNA cleavage sites.

DSBs pose a major threat to genetic integrity and consequently are a leading cause of chromosomal aberrations and cancer in cells^23,24^. Genomic instability results from dysregulation of cell cycle checkpoints or defects in DNA repair^25^ and it has also been reported that ageing induced by oncogenes is part of the barrier to tumorigenesis imposed by the DNA damage checkpoint^26,27^.

In our experiment, apoptosis was not so obviously observed in the position of stem cell, DNA DSB by γ-H2AX expression, also senescence marker, clearly observed in the position of stem cell (stem cell exhaustion). Pyroptosis by AIM2 expression as inflammation marker, was observed in the wide range of positions of stem cell at 2 months after radiation (Fig. 5). Mechanistically, loss of clonogenic (stem/progenitor) cells in the crypts has been suggested to be responsible for radiation-induced intestinal damage^28^. These suggested that internal exposure potentiate senescence in stem cell and pro-inflammatory programmed cell death. That’s geroconversion; it converts reversible arrest to irreversible senescence, which leads to hyper-secretory, hypertrophic and pro-inflammatory cellular phenotypes, hyperfunctions and malfunctions. On organismal level, geroconversion leads to age-related diseases and death^29,30^.

Early event damage to small intestinal tissue for the internal exposure is uncharacteristically rapid and severe compared with external radiation exposure. Whole crypt pyroptosis was not observed in the ^60^Co external exposed group in our study, nor even at the significantly higher dose of 14.2 Gy in Hu et al.^22^. No increase of apoptosis confined to the position of transit cells in the rat small intestine and no decrease of apoptosis, mitosis and AIM2 expressions were observed with the internal exposure compared with external exposure, suggesting a unique radiation pathology.

Intestinal pyroptosis is characterized histologically by the destruction of small intestinal crypt with massive cell death^22^. The pyroptosis proposed by Cookson et al.^31^ represents inflammatory programmed cell death, and activation of the AIM2 inflammasome in response to cytoplasmic DNA　can induce massive caspase-1-dependent cell death and severe damage^20,32^.

Apoptosis and pyroptosis by AIM2 immunohistochemistry were observed in the small intestine of rats at the position of stem cells for both 1× and 4× ^56^Mn internal exposure, with DNA DSB detected by γ-H2AX expression. Taken together, our data suggest that the AIM2 inflammasome mediated pyroptosis of clonogenic cells in the intestinal crypts plays a critical role in the internal radiation-induced GI syndrome. These are similar to models of apoptosis-^28^ and pyroptosis-dependent cell death in the small intestine^22,31^, as well as in high-dose ionizing radiation external exposures^20,22,32^.

Our findings demonstrated that the mechanism of internal radiation injury in the small intestine involves apoptotic and pyroptotic damage at the stem cell position. Apoptosis and pyroptosis was clearly and persistently evident in the small intestinal crypt at the position of stem cell in the ^56^Mn group (^56^Mn(1×);1.33 Gy) until two months later after internal exposure. Moreover, on the 14th day after internal exposure, cytoplasmic AIM2-positive structural abnormal crypts (non-straight and distorted crypts) were formed in the ^56^Mn group (^56^Mn(1×);1.33 Gy), which are not seen in the external exposure (γ2Gy). Crypt structural abnormalities may be induced, triggered by persistent apoptosis and pyroptosis at the position of stem cell in the crypt of the small intestine (stem cell exhaustion), as seen after internal exposure by the ^56^Mn group.

After 3 days, γ2Gy group (small intestinal cell turn-over rate) showed similar low AIM2 expression as the (1) inflammation; ^56^Mn(2×) and^56^Mn(4×) (Fig. 4C, Supplementary Table S1), (2) tissue repair; roughly equivalent effect of mitosis to the ^56^Mn(4×), (3) tissue damage; apoptosis about same level to ^56^Mn(2×) and ^56^Mn(4×), and (4) morphological changes; no crypt shortening, while severe in ^56^Mn(2×) and ^56^Mn(4×), and; no crypt distortion, while observed both in ^56^Mn(2×) and ^56^Mn(4×) at 14 days. These evidences indicate the cause of the severer effect such as inflammation and morphological changes (crypt shortening or crypt distortion) of the internal exposure is likely the varieties of local dose^6^. This is also indicated by the high-LET-like property of internal exposure in the lungs in series of our experiments.γ2Gy group at 2 and 6 months showed slight increasing of apoptosis and pyroptosis, respectively (Fig. 4, Supplementary Table S1), while prominently both in ^56^Mn(2×) and^56^Mn(4×). Man et al. reported that upon dysregulated Wnt signaling, AIM2 suppressed tumor-initiating expansion of intestinal stem cells lining on the base of the crypt^33^. A previous study has shown that over 50% of tumors from patients with small bowel cancer have frameshift mutations in the gene encoding AIM2^34^. Our results may be related to the anti-tumor effects of AIM2, as the both 2× and 4× internal exposures resulted in high score levels for mitosis and pyroptosis, with prominent crypt shortening and crypt distortion at day 3 continued to 8 months. In contrast, there were no pathological findings for mitosis or pyroptosis continued to occur in the γ2Gy group. High levels of mitosis and pyroptosis triggered a unique pathology for internal exposure, such as crypt shortening or crypt distortion.

Abnormal crypts are the earliest morphologically identifiable precancerous lesions in the human colon^35–38^. In the human disease ulcerative colitis (UC), apoptosis and shortened or abnormal crypts are observed; long-term cases of UC are at increased risk of developing colorectal cancer arising from chronically inflamed mucosa^39,40^.

The frequencies of cancer incidence in response to low doses of direct radiation from atomic bombs draw complex non-linear curves, which may reflect the effects of internal exposure^41^.

Among the sources of residual radiation, an understanding of residual radiation from neutron activated soil materials on the ground is particularly important in assessing the risks for people who may have moved to these cities soon after the explosion and inhaled radioactive dust^42–44^. Such people have been reported to suffer from various syndromes, such as gastrointestinal disorders, as well as acute radiation exposure^45^. The incidence rate of acute radiation disease including diarrhea has been reported to be 50%, and Sawada calculated the cumulative effective dose as 1.49 ± 0.38 Gy from the incidence of acute radiation disease for an entrant within 1 km of the hypocenter of Hiroshima on 6 August 1945, immediately after the explosion^5^. Sawada also described for survivors of the bombing beyond 1.5–1.7 km that the incidence of acute radiation disease by the internal exposure due to radioactive fallout are more severe than those from external exposure to primary radiation. Sasaki et al. showed that the average total body absorbed dose can be estimated from the rate of chromosomal aberrations. However, the relation between the estimated dose and distance from the hypocenter cannot be explained by primary external irradiation and it might suggest that some people more than 2.4 km away received radiation from sources other than the primary rays^46^.

The radiation-induced diarrhea observed in A-bomb survivors, as described in Introduction, is consistent with the results of the present experiments. Internal exposure in the experiment involves localized high-dose exposure and severe radiation damage to the small intestine.

Chemical species of inhaled and deposited ^56^Mn particles were identified (Sample2, Sample3 and Sample3-fine) as Mn^2+^ using XANES spectroscopy. A ^56^Mn particle that had a valence of four chemical species as MnO_2_ changed to a valence of two after deposition in small intestine tissue as well as lung tissue^6^. Sample3-fine, being composed largely of Fe and located in a small intestine cavity, is likely to be an ejected blood clot around Mn particle in the process of digestive excretion with food or other matter. In contrast to the standard elementary profile of small intestine, Sample3-fine include a large percentage of Fe likely owing to hemorrhage in the early event. Our findings point to the impact of highly localized early radiation effects of internal exposure and the pathologic chain of events initiated by them, whereas the focus of external radiopathology has primarily been on the long-term effects so exclusively.

Since local ultra-high doses from internal exposure are likely to have effects comparable to those of high LET radiation^6,11,47–49^, which also gives a local ultra-high dose, interphase death, i.e. damaged cells undergoing apoptosis or pyroptosis, may contribute to the pathological progression caused by internal exposure. It is possible that tissue cells severely damaged by external exposure to ^60^Co-γ radiation may turn over with new differentiated cells, as the stem cell damage is less severe^1,18,50,51^. The pathology may therefore gradually lighten. Implanted hot particles emit radiation of varying intensities, from low to very high doses, which can cause extremely serious damage to stem cells that are not affected by low doses from external sources. Stem cells affected by internal exposure resulted in potentially triggering serious pathologies, such as crypt shortening and abnormal crypts (non-straight and distorted crypt precancerous lesions). Tissue surrounding hot particles suffers a variety of radiation injuries resulting from various radiation doses of exposures mixed in close proximity.

## Materials and methods

### Chemicals and radiation

The size distribution of MnO_2_ powder particles used in this experiment is same as that previously reported on Stepanenko et al.^14^. MnO_2_ powder containing ^56^Mn (T1/2 = 2.58 h) was produced by neutron activation of 100 mg ^55^MnO_2_ powder (Rare Metallic Co., Ltd., Japan) at the IVG.1 M (“Baikal-1”) nuclear reactor^52^ using a neutron of an irradiation time of 2000s and fluence of 4 × 10^14^ n/cm^2^ (1×), 4000 s (2×), 8000 s (4×). Briefly, the 100 mg of activated powder with ^56^Mn activities of 2.74 × 10^8^ Bq (^56^Mn(1×)), 2× 2.74 × 10^8^ Bq (^56^Mn(2×)) and 4× 2.74 × 10^8^ Bq (^56^Mn(4×)) was sprayed pneumatically over rats located in an experimental box. γ2Gy group was externally exposed to 2.0 Gy of ^60^Co-γ -ray at a dose rate of 2.6 Gy/min using a Teragam K2 unit (UJP Praha, Praha-Zbraslav, Czech Republic). Control group was unexposed. The initial specific activities (activity per mass) of neutron-activated MnO_2_ powder were 4× higher in ^56^Mn(4×) group and 2× higher in ^56^Mn(2×) group. The average radiation doses were also estimated in the same way as Stepanenko et al.^14^.

### Animals and treatment

Ten-week-old male Wistar rats were purchased from Kazakh Scientific Center of Quarantine and Zoonotic Diseases, Almaty, Kazakhstan. They were housed in plastic cages under climate-controlled conditions at 22 ± 2 °C with a relative humidity of 50% ± 10% and a constant day/night cycle (light 0.700–19.00 h). They were maintained with free access to basal diet and tap water. For the study of 8 months, ^56^Mn(1×) rats (3 days; *n* = 4, 14 days; *n* = 4, 2 months; *n* = 4, 3 months; *n* = 4, 8 months; *n* = 8) were compared with a group of rats exposed to Mn-stable (not activated) (3 days; *n* = 4, 14 days; *n* = 4, 2 months; *n* = 4, 3 months; *n* = 4, 8 months; *n* = 8), a group externally exposed to 2.0 Gy ^60^Co γ-ray (3 days; *n* = 4, 14 days; *n* = 4, 2 months; *n* = 4, 3 months; *n* = 4, 8 months; *n* = 8) and an unexposed control group (3 days; *n* = 4, 14 days; *n* = 4, 2 months; *n* = 4, 3 months; *n* = 4, 8 months; *n* = 8) as in^11^.

Pathologic small intestinal samples of ^56^Mn(2×) group (6 h; *n* = 3, 3 days; *n* = 3, 14 days; *n* = 3, 2 months; *n* = 3, 6 months; *n* = 3) and ^56^Mn(4×) group (6 h; *n* = 3, 3 days; *n* = 3, 14 days; *n* = 3, 2 months; *n* = 3, 6 months; *n* = 3), externally exposed to 2.0 Gy ^60^Co γ-ray group (6 h; *n* = 3, 3 days; *n* = 3, 14 days; *n* = 3, 2 months; *n* = 3, 6 months; *n* = 3) and an unexposed control group (6 h; *n* = 3, 3 days; *n* = 3, 14 days; *n* = 3, 2 months; *n* = 3, 6 months; *n* = 3) were observed at 6 h, 3 days,14 days (early phase), 2 months and 6 months (late phase), and 8 months in ^56^Mn(1×) group (late phase) after exposure. There were 3–6 rats in each group.

### Pathology

Small intestines, middle portion, were collected, dissected and fixed in 10% neutral buffered formaldehyde and embedded in paraffin. Sections of 4 μm thickness were prepared and stained with H&E. For pathologic examination of the small intestinal tissue, the number of apoptotic cells and mitotic cells per crypt were counted as in^10,11^. 20 longitudinal crypt sections per mouse were selected and counted. Sections were used for counting the number of mitotic figures with hematoxylin and H&E stain^18^. Ratio of crypt vs. villous length per crypts of the small intestine in rats were measured as in^18,19^, and were analyzed by Olympus cellSens Dimension using 5 images per sample. Briefly, villus length, defined as the length from the apex of the brush border to the base of the crypt in the small intestine, and crypt depth (along the long axis of the elliptical crypt) were measured using a 100× magnification stage micrometer. The lengths of more than five random villi or crypts were measured (Supplementary Table S2), and the measurements were averaged^18^.

The expression of AIM2 (bs-5986R, Bioss Antibodies) and γ-H2AX at (Ser139) (Cell Signaling Technology, Danvers, MA, USA) proteins in small intestinal tissues was assessed using immunohistochemistry. Briefly, paraffin sections were deparaffinized and pretreated with microwave heating for antigen retrieval in 0.01 mol/l citrate buffer (pH 6.0). The sections were reacted with 0.3% H_2_O_2_ in deionized water for 10 min to inhibit endogenous peroxidase activity and incubated with antibodies overnight. After washing with PBS, the sections were incubated for 30 min using an LSAB-2 system-HRP for use on rat specimens (Dako). Antibody binding was visualized by incubation with 3,3’-diaminobenzidine (DAB) chromogen (Dako) according to the manufacturer’s instructions. Apoptotic cells were stained by TUNEL method (ApopTag Peroxidase In Situ Apoptosis Detection Kit, S7100, Chemicon) as described, by the manufactures’ suggested protocol as in^19^. Corresponding serial sections were stained with H&E, γ-H2AX and AIM2 immunohistochemistry, and TUNEL method.

### Quantification of the histological data

The sum of apotosis + (TUNEL positive) cells and Mitosis + cells by H&E stain per crypt in the small intestinal tissues was counted, and the lengths of villi or crypts were measured by two independent observers (Fig. 4).

### SR-XRF-XANES analysis

XRF analyses combined with XANES spectroscopy were performed at BL-4A of the Photon Factory in the High Energy Accelerator Research Organization (KEK-PF, Tsukuba, Japan) as in^6^. All the experiments were carried out in the top-up running mode of PF at room temperature in the ambient air, and the basic processes of the analysis are similar to those described in Takahashi et al.^53^. The energy of the incident X-ray was 12.9 keV. The X-ray was focused into 5 μm (horizontal) × 5 μm (vertical) at BL-4A using Kirkpatrick-Baez mirror optics. A thin-section sample of the specimens was fixed on a sample holder oriented at 45° to the X-ray beam. SR-XRF-XANES was performed on three samples of the ^56^Mn (2×) group. The other group, ^56^Mn (4×), showed that apoptosis had already occurred and the crypt/epithelium ratio was reduced after 6 h, as shown in Fig. 4A,D. This was due to the fact that the tissue damage had progressed and the results of SR-XRF-XANES were not obtained. This means that tissue damage had progressed and the SR-XRF-XANES results were not obtained. The sample was irradiated by the micro-focused X-ray and the specimen stage was scanned in the X–Y directions, two-dimensionally, to obtain the areal elemental distribution images using intensities of XRF from each element detected by a silicon drift detector (SDD). The size of the scanned areas varied within several millimeters and the scanning steps varied from 5 to 50 μm. The obtained XRF spectra at BL-4A were processed using PyMca software (Version 4.7.3). In addition, the XRF spectra were measured for 300 s at the spots containing metallic elements as in^6^.

Chemical species of Mn such as valence of Mn at the point of interest (POI) found by SR-XRF analysis was determined by Mn K-edge XANES spectroscopy^54^. The uncertainty in energy is 0.1 eV. The spectra at the POI were compared with those of reference compounds including MnO_2_, Mn_2_O_3_, Mn_3_O_4_ and MnCO_3_ to estimate average valence of Mn at the POI. The XANES spectra of Mn were processed using a XAFS data analysis software, REX2000 (Rigaku Co. Tokyo, Japan) as in^6^.

### Statistical analysis

All values were expressed as the mean ± standard error (SE) involving three to six animals. Mann–Whitney U test was applied to evaluate the statistical significance of difference between groups^55^. All statistical tests were performed using Microsoft Excel 2016 (Microsoft Corporation, Redmond, WA, USA) and the add-in software Statcel3.

### Ethical approval

All applicable international, national, and/or institutional guidelines for the care and use of animals were followed. The animal experiment was approved by the Animal Experiment committee of Semey Medical University, Republic of Kazakhstan (Protocol No. 5 dated 16 April 2014) and conducted in accordance with the Institutional Guide for Animal Care and Use. The authors complied with the Journal’s position on issues involved in ethical publication (the Arrive guidelines 2.0).

## Supplementary Information

Supplementary Information.

## Data Availability

All data generated or analyzed during this study are included in this article.
